# BCL-3 promotes the tumor growth of hepatocellular carcinoma by regulating cell proliferation and the cell cycle through cyclin D1

**DOI:** 10.3892/or.2022.8456

**Published:** 2022-12-01

**Authors:** Kangsheng Tu, Zhikui Liu, Bowen Yao, Yumo Xue, Meng Xu, Changwei Dou, Guozhi Yin, Jun Wang

Oncol Rep 35: 2382–2390, 2016; DOI: 10.3892/or.2016.4616

Subsequently to the publication of the above article, the authors have alerted the Editorial Office to the fact that they identified a small number of errors concerning the assembly of Figs. 3A, 6B and 7A in their paper. Specifically, the western blotting results for the BCL-3 and GAPDH experiments in Fig. 3A, the cyclin D1 blots in Fig. 6B and the cyclin D1 blots shown in Fig. 7A were selected erroneously when choosing images from the total pool of data due to the similarity in the appearance of the data. However, the authors retained their access to the raw data, and were able to make the appropriate corrections required for these figures.

The corrected versions of Figs. 3, 6 and 7, showing the correct BLC-3/GAPDH and cyclin D1 data in Fig. 3A and 6B respectively, and the correct cyclin D1 data in Fig. 7A, are shown on the next two pages. Note that these errors did not adversely affect the major conclusions reported in the study. The authors all agree to the publication of this corrigendum, and thank the Editor of *Oncology Reports* for allowing them the opportunity to publish this. The authors also apologize for any inconvenience caused.

## Figures and Tables

**Figure 3.**
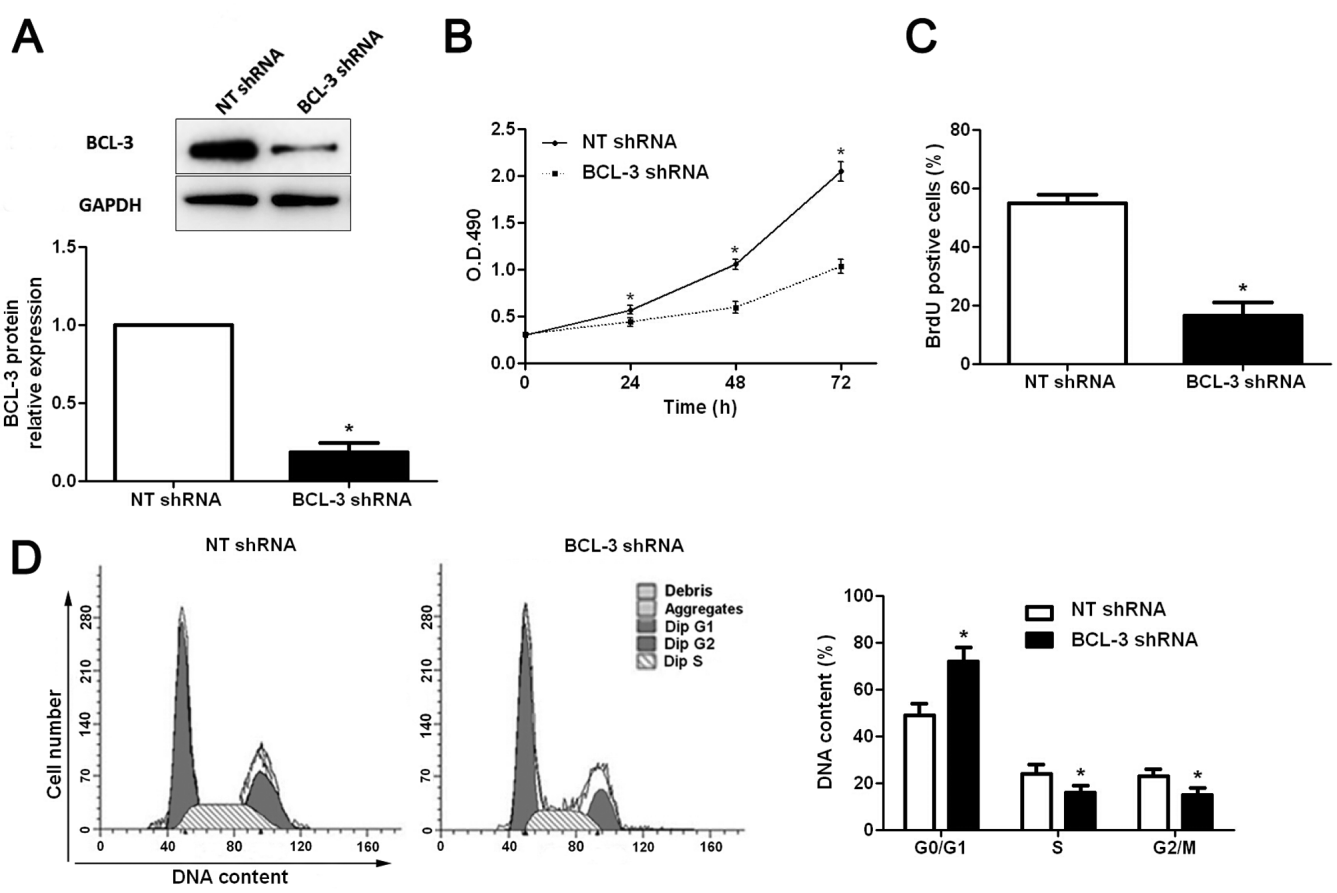
Suppression of BCL-3 expression inhibits the cell viability, proliferation and cell cycle progression in HepG2 cells. (A) BCL-3-specific shRNA significantly inhibited the levels of BCL-3 protein in the HepG2 cells. n=3 independent experiments. *P<0.05 by t-test. (B) Cell viability of HepG2 cells as assessed by MTT assays was suppressed by BCL-3 silencing. n=3 independent experiments. *P<0.05 by ANOVA. (C) Cell proliferation as assessed by BrdU incorporation was inhibited after BCL-3 knockdown. n=3 independent experiments. *P<0.05 by t-test. (D) Cell cycle assays demonstrated that BCL-3 knockdown increased the percentage of HepG2 cells in the G0/G1 phase while decreased the percentage of HepG2 cells in the S and G2/M phase. n=3 independent repeats with similar results. *P<0.05 by t-test.

**Figure 6.**
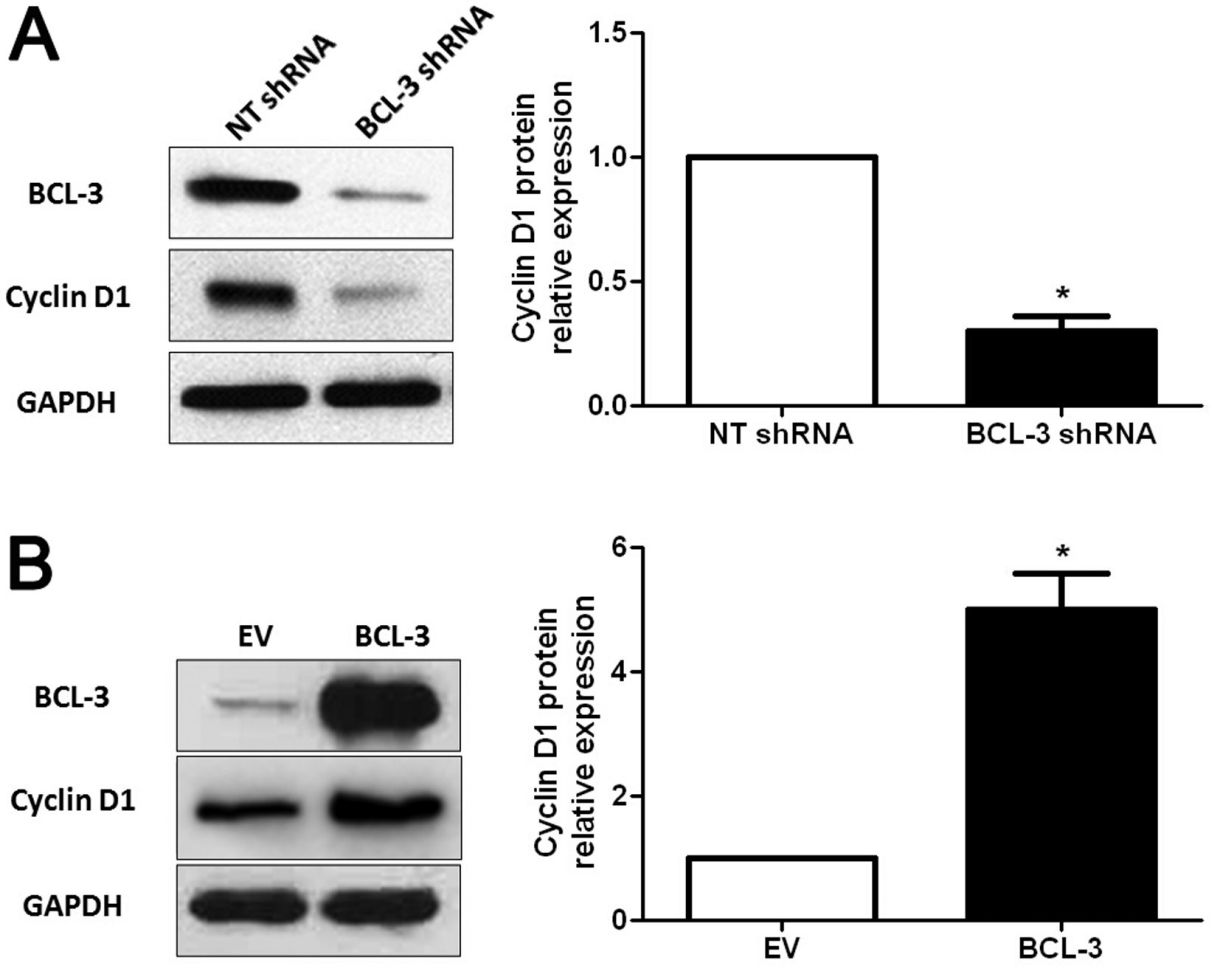
BCL-3 regulates the expression of cyclin D1 in HCC cells. (A) BCL-3 knockdown in HepG2 cells significantly reduced the protein level of cyclin D1. n=3 independent repeats with similar results; *P<0.05 by t-test. (B) Overexpression of BCL-3 obviously increased the expression of cyclin D1 protein in Huh7 cells. n=3 independent repeats with similar results; *P<0.05 by t-test.

**Figure 7.**
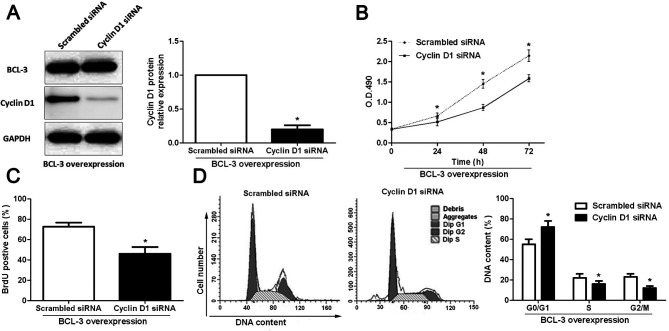
Inhibition of cyclin D1 expression abrogates the effect of BCL-3 on Huh7 cells. (A) Cyclin D1-specific siRNA significantly inhibited the expression of cyclin D1 while had no influence on BCL-3 in the BCL-3-overexpressing Huh7 cells. n=3 repeats with similar results; *P<0.05 by t-test. Inhibition of cyclin D1 expression abolished the functional effect of BCL-3 on (B) cell viability, (C) proliferation and (D) cell cycle progression. n=3 repeats with similar results; *P<0.05 by ANOVA (MTT assay) and t-test (BrdU incorporation and cell cycle assays).

